# The Flavor Characteristics, Antioxidant Capability, and Storage Year Discrimination Based on Backpropagation Neural Network of Organic Green Tea (*Camellia sinensis*) during Long-Term Storage

**DOI:** 10.3390/foods13050753

**Published:** 2024-02-29

**Authors:** Xiaomei Wen, Shanjie Han, Jiahui Wang, Yanxia Zhang, Lining Tan, Chen Chen, Baoyu Han, Mengxin Wang

**Affiliations:** 1 Zhejiang Provincial Key Laboratory of Biometrology and Inspection and Quarantine, College of Life Sciences, China Jiliang University, Hangzhou 310018, China; s21090710057@cjlu.edu.cn (X.W.); hanshanjie@126.com (S.H.); 2000910117@cjlu.edu.cn (J.W.); 2000910116@cjlu.edu.cn (Y.Z.); 2000910107@cjlu.edu.cn (L.T.); 2000910108@cjlu.edu.cn (C.C.); hanbaoyu@cjlu.edu.cn (B.H.); 2 Hangzhou Tea & Chrysanthemum Technology, Co., Ltd., Hangzhou 310018, China

**Keywords:** organic green tea, storage year, functional components, electronic tongue, classified prediction model

## Abstract

The storage period of tea is a major factor affecting tea quality. However, the effect of storage years on the non-volatile major functional components and quality of green tea remains largely unknown. In this study, a comparative analysis of organic green teas with varying storage years (1–16 years) was conducted by quantifying 47 functional components, using electronic tongue and chromatic aberration technology, alongside an evaluation of antioxidative capacity. The results indicated a significant negative correlation between the storage years and levels of tea polyphenols, total amino acids, soluble sugars, two phenolic acids, four flavonols, three tea pigments, umami amino acids, and sweet amino acids. The multivariate statistical analysis revealed that 10 functional components were identified as effective in distinguishing organic green teas with different storage years. Electronic tongue technology categorized organic green teas with different storage years into three classes. The backpropagation neural network (BPNN) analysis demonstrated that the classification predictive ability of the model based on the electronic tongue was superior to the one based on color difference values and 10 functional components. The combined analysis of antioxidative activity and functional components suggested that organic green teas with shorter storage periods exhibited stronger abilities to suppress superoxide anion radicals and hydroxyl radicals and reduce iron ions due to the higher content of eight components. Long-term-stored organic green teas, with a higher content of substances like L-serine and theabrownins, demonstrated stronger antioxidative capabilities in clearing both lipid-soluble and water-soluble free radicals. Therefore, this study provided a theoretical basis for the quality assessment of green tea and prediction of green tea storage periods.

## 1. Introduction

Tea is one of the most popular and healthy beverages in the world, all derived from the processed young tea shoots of *Camellia sinensis*. Based on processing technology and fermentation extent, Chinese teas can be categorized into six basic tea types recognized as green tea, white tea, yellow tea, oolong tea, black tea, and dark tea. Green tea is the most produced and consumed type of tea in China. Its popularity is attributed not only to the fragrant taste, but also to the various health benefits associated with its active ingredients, such as antioxidant, anti-inflammatory, blood-sugar-lowering, and preventive effects against cardiovascular diseases and cancer [1,2,3,4]. When the production of green tea exceeds market demand, some green tea products inevitably remain stored in warehouses for a certain period. Studies have reported that two-thirds of stored tea leaves are green tea, with organic green tea accounting for 7% of the total green tea volume [5]. In comparison to regular teas, organic green tea contains more functional compounds, offering higher health benefits [6]. As an unfermented tea, the functional components and quality of green tea may undergo significant changes during prolonged storage, affecting its quality and nutritional value.

The influence of storage year on tea quality is primarily reflected in the changes in key compounds, impacting tea color, aroma, taste, and antioxidative capabilities. Research on the changes in non-volatile compounds during long-term storage by year has mainly focused on white tea, black tea, oolong tea, and dark tea. In the aging process of Jinhua white tea over 5–10 years, the content of tea polyphenols, catechins, caffeine, and amino acids significantly decreased, while gallic acid and theabrownins increased [7]. Long-term storage of large-leaf black tea resulted in a decrease in theaflavins, catechins, and amino acids, and an increase in theabrownins and thearubigins [8]. Lipids and organic acids in oolong tea increased with storage years, while tannins, phenolic acids, amino acids, and their derivatives decreased [9]. For Qingzhuan tea, the 10th year was a critical period, with the highest content of tea polyphenols and total flavonoids, and an increase in theabrownins in the later years [10]. Research on green tea during long-term storage has mainly focused on changes in volatile compounds. Liu et al. [11] preliminarily identified the key stages of quality changes in Meixian green tea during storage by the changes in 11 volatile compounds. Our previous studies have identified six key volatile compounds for organic green tea during long-term storage [5]. However, the dynamic changes in non-volatile major functional components and the quality of green tea during long-term storage remain largely unknown. *N*-ethyl-2-pyrrolidinone-substituted flavan-3-ols have been identified as a marker compound related to the storage of green tea within 19 months [12]. Therefore, a thorough analysis of the changes in the major functional components, taste, color quality, and antioxidative activity of organic green tea during long-term storage will facilitate the understanding of the impact of storage on the quality and nutritional value of green tea.

Artificial neural networks (ANNs) have been extensively used in the food industry. Due to their non-parametric nature, artificial neural networks can effectively simulate the nonlinear changes in quality components during food storage and are commonly used to predict food quality and shelf life. Therefore, the backpropagation neural network (BPNN) model is one of the most widely used neural network models for predicting food shelf life [13]. Studies on predicting the storage period of non-volatile compounds in tea have mainly focused on white tea, black tea, and dark tea [14,15,16], while research on constructing storage period discrimination models for green tea stored for one year is mainly based on electronic tongue or near-infrared hyperspectral analyses [17,18]. However, there is currently relatively little research on constructing storage period discrimination models based on the content of major non-volatile functional components in green tea during long-term storage. Therefore, building a discrimination model based on non-volatile compounds and intelligent sensing of signals of organic green tea can more accurately assess the shelf life of green tea.

To reveal the dynamics of flavor characteristics and antioxidative activity of organic green tea during long-term storage and the correlation between storage years and key non-volatile compounds, this study comprehensively characterized organic green tea stored continuously for 1–16 years through multiple approaches, including quantitative analysis of the main chemical components, intelligent sensory analysis (electronic tongue and colorimeter), and in vitro antioxidant activity analysis. Additionally, a classification prediction model for organic green tea with varied storage years was constructed based on the change in the BPNN level. This provides new insights into the key non-volatile compounds affecting the quality of organic green tea and the evaluation of green tea storage periods, aiding in a better understanding of the impact of storage on the quality of green tea.

## 2. Materials and Methods

### 2.1. Tea Samples and Chemicals

Organic green teas stored for 1–16 years were collected from the mountain range with a height of 450 to 500 m of Zhejiang Meifeng Tea Industry Co., Ltd. in Liandu District, Lishui City, China (location 28°38′38″ N, 119°57′53″ E), and they were produced annually from 2006 to 2021 and retained their original packaging and detailed labels, providing comprehensive sample information. All samples were harvested from the same tea plant cultivar (*Camellia sinensis* cv. ‘Longjing 43’) at the same location during the same season (pure brightness in spring) and processed using identical techniques (local traditional green tea processing technology such as fixation, rolling and drying) to ensure as similar initial chemical compositions as possible for more accurate results. Detailed information about the samples is available in Table S1. After preparation, all tea samples were stored in the same environment (dry and ventilated, ≤50% relative humidity) in a warehouse under natural conditions. Each organic green tea sample was collected from three different production batches and stored at −20 °C for further analysis.

Standards of caffeine, catechin (C), epicatechin (EC), epigallocatechin (EGC), gallocatechin (GC), epicatechin gallate (ECG), epigallocatechin gallate (EGCG), gallocatechin gallate (GCG), catechin gallate (CG), gallic acid (GA), and ellagic acid were procured form Macklin Biochemical Technology Co., Ltd. (Shanghai, China). Seven flavonols standards of taxifolin, luteolin, myricetin, kaempferol, quercetin, luteoloside, and rutin were purchased from Aladdin Biochemical Technology Co., Ltd. (Shanghai, China). Eighteen amino acid standards of L-theanine, L-aspartic acid, L-glutamic acid, L-serine, glycine, L-histidine, L-arginine, L-threonine, L-alanine, L-proline, L-cystine, L-tyrosine, L-valine, L-methionine, L-isoleucine, L-leucine, L-phenylalanine, L-lysine were purchased from Agilent Technologies Co., Ltd. (Shanghai, China). The purity of these standards was over 98%. Chromatographic grade acetonitrile, methanol, and formic acid were provided by Tedia Co., Inc. (Fairfield, OH, USA). All other chemicals were of the highest analytical grade.

### 2.2. Determinations of Main Quality Components in Organic Green Tea

Adhering to national standards (GB/T 8313-2018) [19], the quantification of tea polyphenols at 765 nm was conducted using the Folin–Ciocalteu method with gallic acid as the standard, employing a UV-1100 ultraviolet-visible (UV-vis) spectrophotometer (Mapada, Shanghai, China). The total flavonoid content at 510 nm was determined using rutin as the standard. For assessing soluble sugar content, glucose was utilized as the standard, and the anthrone method at 620 nm was employed. According to national standards (GB/T 8314-2013) [20], L-glutamine served as the standard for measuring total amino acid content through a ninhydrin assay at 570 nm. The contents of theaflavins (TFs), thearubigins (TRs), and theabrownins (TBs) were analyzed by spectrophotometry according to Zhu et al. [21]. The anthocyanins content of the organic green tea samples was determined by ultraviolet spectrophotometer following the procedure of Amulya and Islam [22]. The chlorophyll-and total carotenoids of the organic green tea samples were extracted by the method of Xie et al. [23] and measured at 470, 645, and 662 nm to quantify the chlorophyll-and total carotenoids contents.

The contents of caffeine, GA, and eight catechins (GC, EGC, C, EC, EGCG, GCG, ECG, CG) in organic green tea samples were determined using an Agilent 1260 series high-performance liquid chromatography (HPLC) system (Agilent Technologies, Santa Clara, CA, USA) according the previous method used in our research [24] and GB/T 8313-2018 [19] with slight modifications. A Phenomenex/Luna C18 column (250 mm × 4.6 mm, 5 μm; Phenomenex, Torrance, CA, USA) was used. The mobile phase A was 9% acetonitrile, 2% acetic acid, and 0.2% EDTA-2Na solution, and the mobile phase B was 80% acetonitrile, 2% acetic acid, and 0.2% EDTA-2Na solution. The gradient was programmed as follows: the gradient mobile phase A was 100%, 0–10 min; 100–68%, 10–15 min; 68%, 15–25 min; 68–100%, 25–30 min; 100%, 30–35 min. The flow rate was 1 mL/min, the detection wavelength was 278 nm, and the injection volume was 20 μL.

The contents of eighteen amino acids, ellagic acid, and seven flavonoids in organic green tea samples were analyzed by the established and evaluated HPLC methods [25,26] using the instruments and chromatographic columns described above. The chromatographic conditions for detecting eighteen free amino acids were as follows. The detection wavelength was 254 nm and the column temperature was set at 43 °C. The flow rate was 1.0 mL/min. The injection volume was 20 μL. The mobile phases A (930 mL 0.l mol/L sodium acetate buffer and 70 mL acetonitrile solution) and B (80% acetonitrile solution) were prepared for HPLC. The gradient mobile phase B was 0–4%, 0–20 min; 4–20%, 20–21 min; 20–22%, 21–40 min; 22–30%, 40–41 min; 30–100%, 41–46 min; 100%, 46–48 min; 100–0%, 48–53 min; 0%, 53–60 min. The chromatographic conditions for detecting ellagic acid and seven flavonoids were as follows. The flow rate was 0.8 mL/min and the detection wavelength was 370 nm. The gradient mobile phase B was 10–45%, 0–16 min; 45–65%, 16–22 min; 65–100%, 22–25.9 min; 100–10%, 25.9–29 min; 10%, 30–36 min. All components were tested using three biological replicates.

### 2.3. Intelligent Sensory Evaluation

#### 2.3.1. Electronic Tongue Measurement

The electronic tongue mimics the human tongue in analyzing the taste of food, enabling a quick and objective assessment of the overall taste and quality information of the sample [27]. In this study, the taste of organic green teas from different storage years was evaluated with an Astree electronic tongue (Alpha MOS, Toulouse, France). The electronic tongue consists of 7 sensors (SRS, STS, SWS, UMS, BRS, SPS, GPS) and a Ag/AgCl reference electrode, with sensors specifically responding to five basic tastes: sourness (SRS), sweetness (SWS), bitterness (BRS), saltiness (STS), and umami (UMS). Approximately 3.0 g of organic green tea sample was placed in a conical flask, 150 mL of boiling distilled water was added, and after 5 min of steeping, the mixture was filtered. The filtrate, cooled to room temperature, was used for electronic tongue analysis. Prior to data collection, the system underwent autotesting, conditioning, calibration, and diagnostic procedures, ensuring the reliability and stability of sensor response signals. Data collection time was set at 120 s, stirring rate at 1 cycle/s, and the average value of the last 20 s measurements was taken as the response value for each sensor. After each sampling, the sensors underwent a 10 s rinse in the cleaning solution to avoid any impact on the next sampling. Each sample was repeated 12 times.

#### 2.3.2. Colorimeter Measurement

Color parameters of both organic green tea leaves and infusions were measured with a CM-3600d colorimeter (Konica Minolta, Shanghai, China) using the Lab color system. The appearance color of tea samples was measured directly. The tea infusions were prepared according to the national standard (GB/T 23776-2018) [28]. Approximately 3.0 g of organic green tea was sampled, 150 mL of boiling distilled water was added, and after 5 min of steeping, the tea infusion was filtered. The color parameters (L*, a*, b*) were then measured after the filtrate cooled to room temperature. L* indicates brightness, with values ranging from 0 to 100, where higher values represent higher brightness. The a* value represents red-green color, with “+” indicating red and “-” indicating green. The b* value represents yellow-blue color, with “+” indicating yellow and “-” indicating blue. ΔE denotes the total color difference [21]. Measurement of each sample was repeated 9 times.

### 2.4. Determination of In Vitro Antioxidant Activities

The antioxidative properties of organic green teas were evaluated through five in vitro assays, namely 2,2-Diphenyl-1-picrylhydrazyl free radical scavenging capacity (DPPH), superoxide anion scavenging capacity (SSA), hydroxy free radical scavenging activity (HSA), ferrous ion reduction capacity (FRAP), and ABTS·+ reducing capacity (ABTS) [29]. Specifically, 0.1× *g* of the organic green tea sample was blended with 1 mL of an 80% methanol solution (or 0.9 mL physiological saline), homogenized in an ice-water bath, and following centrifugation, the resulting supernatant was utilized for DPPH (or ABTS) detection. For SSA and HSA detection, 0.1× *g* of the organic green tea sample was mixed with 5 mL of distilled water, boiled for 10 min, and after centrifugation, the supernatant was collected. In the case of FRAP detection, 1× *g* of the organic green tea sample was combined with 4 mL of physiological saline, homogenized in an ice-water bath, and post-centrifugation, the supernatant was employed for analysis. All antioxidant indices were tested using three biological replicates. The specific calculation methods for the 5 antioxidant activities are shown in the Supplementary Materials.

### 2.5. The Construction of a Classification Prediction Model Based on BPNN

The BPNN is currently the dominant artificial neural network architecture used for food quality and storage period prediction [13]. A BPNN consists of an input layer, one or more hidden layers, and an output layer, each of which may contain multiple nodes. In each node, an activation function is used to introduce a nonlinear transformation. Common activation functions include sigmoid, tansig, and Gaussian functions, which enable neural networks to learn nonlinear relationships. The training process of a BPNN uses the backpropagation algorithm, which adjusts the network weights to reduce the error by calculating the error between the network’s outputs and the actual target and propagating this error backward (Figure S1).

Before the model was built, the data in each dataset was normalized to eliminate the effect of indicator dimensions. In order to improve discrimination accuracy, all the samples were randomly divided into training and test sets, wherein 75% of the samples were divided into the training set and 25% of the samples were divided into the test set. The BPNN was used to construct a classification prediction model for the storage time of organic green teas. This program employed the ‘tansig’ function as the activation function for the hidden layer and the ‘softmax’ function for the output layer. The Levenberg–Marquardt backpropagation algorithm, specifically the ‘trainlm’ function, was used to train the neural network. Training ceased when the maximum iteration count reached 1000, and the learning rate was set at 0.01.

When building the network model, the number of neurons in the input layer is the same as the dimension of the input data, and the number of neurons in the output layer is the same as the number of data to be fitted. Mean square error (MSE) and the correlation coefficient R are crucial parameters for evaluating the performance of the network model. The smaller the MSE, the better the network model performance. The network model should select the number of hidden-layer neurons corresponding to the minimum MSE based on the training data. The correlation coefficient R evaluates the linear correlation between the actual and predicted values. The larger the correlation coefficient R, the better the network model performance. The minimum MSE and maximum R correspond to the optimal network model [30,31].

$$MSE=\frac{1}{n}\sum_{i=1}^{n}{\left(X_{i}-Y_{i}\right)}^{2}$$

$$R=\frac{\sum_{i=1}^{n}\left(X_{i}-X\right)\left(Y_{i}-Y\right)}{\sum_{i=1}^{n}{\left(X_{i}-X\right)}^{2}\sum_{i=1}^{n}{\left(Y_{i}-Y\right)}^{2}}$$

where *n* is the number of samples, *X_i_* is the actual value of the neural network, *Y_i_* is the predicted value of the neural network, *X* is the average of the actual value of the neural network, and *Y* is the average of the predicted value of the neural network.

### 2.6. Statistical Analysis

Data are expressed as mean values ± standard deviation. One-way analysis of variance (ANOVA), non-parametric test (Kruskal–Wallis test) and Pearson correlation analysis were performed with IBM SPSS 24.0 software (Chicago, IL, USA). Multivariate statistical analysis was performed with SIMCA 14.1 software (Umetrics, Umea, Sweden). Principal component analysis (PCA), hierarchical cluster analysis (HCA), and bar charts were produced using Origin Pro 2021 software (Hampton, VA, USA). Heat maps were plotted through TBtools. BPNN models were performed using MATLAB software (version 2020b). Correlation network plots were drawn using Cytoscape (version 3.9.0, Beijing, China).

## 3. Results and Discussion

### 3.1. Dynamic Changes in Functional Components of Organic Green Tea during Storage

#### 3.1.1. Comparative Analysis for the Contents of Biochemical Components of Organic Green Tea during Storage

**Figure 1:**
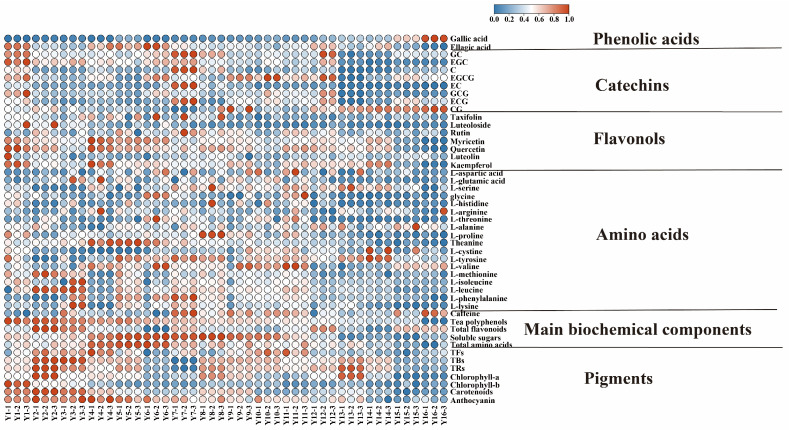
The variation in chemical components during long-term storage of organic green tea.

The results of the analysis of the biochemical composition of organic green tea with different storage years are shown in Figure 1 and Table S2, which to some extent can reflect the change trend of the taste of organic green tea in the storage process. Due to the use of the same tea plant cultivars, planting locations, processing methods, and storage environment, the changes in biochemical composition were mainly influenced by the storage year.

Tea polyphenol is a key component of green tea, impacting not only the color and flavor of the tea but also playing an indirect role in the alterations of other chemical constituents [32]. Throughout the storage of the organic green teas, there was a consistent and noteworthy decline in polyphenol content (*p* < 0.01). This result is consistent with the overall decreasing pattern of polyphenols seen in the prolonged storage of white tea [33]. Amino acids serve as the primary flavor-contributing compounds in tea, playing a crucial role in the taste and aroma. The total amino acid content demonstrated a dynamic pattern characterized by an initial increase followed by a subsequent decrease during the storage period, revealing highly significant variations related to storage time (*p* < 0.01). The likely explanation for this phenomenon lies in the hydrolysis of water-soluble proteins in the early stages of storage, leading to the accumulation of amino acids and a marginal increase in their total quantity. However, as storage time extended, oxidative reactions, degradation, and transformations of amino acids took place, such as the Strecker and Maillard reactions [34], resulting in a substantial decrease in the total amino acid content. This finding aligns with the transformations observed in Liupao tea [35] and large-leaf black tea [8] throughout the storage process. Soluble sugar, a primary sweet component in tea, can mitigate the bitter taste associated with certain substances in tea infusion. The higher the content of soluble sugars, the more sweet and mellow the taste of tea [4,36]. The soluble sugar content exhibited an initial upward trend followed by a subsequent decline during the storage time. A marginal increase was noticeable during the 5–8 years of storage, although this difference lacked statistical significance. However, a notable decrease became evident afterward, indicating that the soluble sugar content in organic green tea experiences a slight elevation during a specific storage period. This trend was consistent with the variations observed in Wuyi rock tea across different storage years [37]. In the context of long-term storage, a significant reduction occurred, aligning with the observed decline in sweetness in organic green teas. Flavonoids play a pivotal role in assessing the quality of tea. Throughout the storage of the organic green teas, the total flavonoid content exhibited a fluctuating trend. Notably, the total flavonoid content of tea samples stored for 16 years increased by 14.83%, compared to those stored for 1 year. Previous studies have reported that as storage time increased, the total flavonoid content in white tea experienced a significant rise [33]. In the case of Qingzhuan tea, the total flavonoid content remained relatively steady during the initial 1–5 years of storage but achieved its peak after 10 years [10]. Caffeine, a fundamental flavor compound in tea, forms complexes through hydrogen bonding with catechins, contributing to a fresh taste [32]. While there was a slight increase in caffeine during the seventh year of organic green tea storage, its variation was relatively mild over prolonged storage, maintaining a relatively stable content. Our findings align with prior research, demonstrating the stability of caffeine content during the natural storage processes of both green and dark teas [10,12].

**Figure 2:**
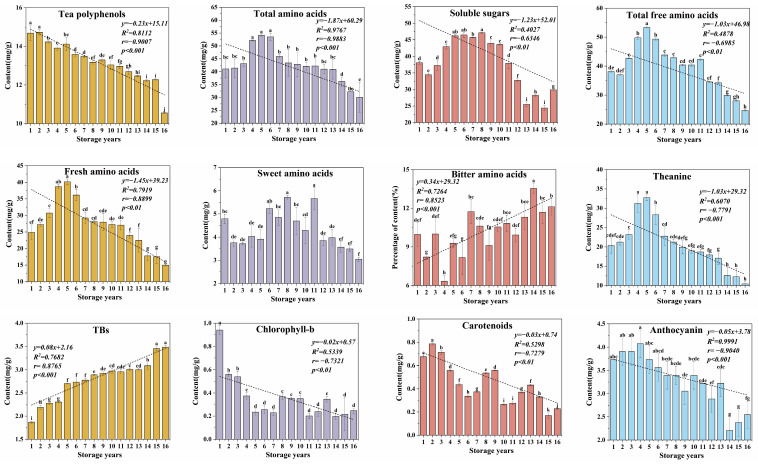
The typical patterns of change in the representative components of organic green tea during long-term storage. Different letters represent significance at the level of *p* < 0.05.

Correlation analyses (Figure 2) revealed notable patterns: while total flavonoids and caffeine exhibit distinct relationships, a robust linear correlation exists between tea polyphenols and storage years (r = −0.9007, *p* < 0.001). Additionally, a pronounced negative correlation was observed between total amino acid content and storage years (r = −0.9883, *p* < 0.001), accompanied by a significant negative correlation between soluble sugar and storage years (r = −0.6346, *p* < 0.01). These identified correlations underscore the significance of tea polyphenols, total amino acids, and soluble sugar as crucial indicators for evaluating the storage time of green tea.

#### 3.1.2. Changes in Catechins and Gallic Acid of Organic Green Tea during Storage

Catechins and gallic acid are important components in the secondary metabolism of tea plants, and are also the primary components of the health functions of tea, and play an important role in the formation of the color and flavor qualities of tea. The catechins in tea are mainly gallocatechin (GC), catechin (C), catechin gallate (CG), epicatechin gallate (ECG), gallocatechin gallate (GCG), epigallocatechin (EGC), epicatechin (EC), and epigallocatechin gallate (EGCG), which are classified into galloylated catechins and nongalloylated catechins, with galloylated catechins including CG, GCG, ECG and EGCG, and nongalloylated catechins including C, EC, GC, and EGC [12,38]. Catechins are the most abundant compounds in organic green teas and the primary contributors to the antioxidative properties of the teas. Following 16 years of storage, the contents of ECG, EGCG, C, GC, EGC, EC, and GCG decreased by 28%, 36%, 36%, 52%, 62%, 73%, and 76%, respectively. Conversely, CG content exhibited a significant increase (*p* < 0.05) with each passing year of storage. Interestingly, nongalloylated catechins were more prone to decline than galloylated catechins, consistent with the findings of Dai et al. [12]. EGCG and ECG are the most abundant compounds of catechins in organic green tea, which guide the changes in catechins contents. The trends of change in EGCG and ECG during storage are similar, with a significant continuous decrease in their content during the first 1–3 years of storage, followed by fluctuating changes, influencing the overall trend in catechins (Figure 1). Previous studies have confirmed that the decrease in catechins during storage was mainly through degradation, autoxidation, or reactions with other components [39]. In the storage process of white tea, catechins formed seven novel 8-C *N*-ethyl-2-pyrrolidinonesubstituted flavanol (EPSF) compounds with theanine [14]. EGCG is the predominant catechin in organic green tea, and the decline in catechins is primarily manifested through the conversion of EGCG. EGCG can undergo degradation, transforming into gallic acid, and binding with theanine to generate EGCG-cThea [40]. EGCG can also autoxidize into dimeric catechins, such as theaflavins and polymerized catechins. Gallic acid is a natural antioxidant with anti-tumor properties [41]. Gallic acid is primarily produced by the hydrolysis of galloylated catechins. In the long-term storage process of organic green teas, the content of gallic acid significantly increased (*p* < 0.05), reaching its lowest point in tea samples stored for 1 year and the highest in tea samples stored for 16 years. This suggests that in the storage process of organic green teas, the hydrolysis of galloylated catechins exceeds the degradation of gallic acid, providing a basis for the transformation of catechins.

#### 3.1.3. Changes in Flavonols and Ellagic Acid of Organic Green Tea during Storage

Flavonol compounds, such as kaempferol, quercetin, myricetin, etc., are significant tea polyphenols in green tea, with contents ranking second only to flavanols. These compounds play a certain role in influencing the color and taste of green tea liquor. In the storage of organic green teas, the overall content of flavonol compounds exhibited a significant declining pattern (*p* < 0.05) (Figure 1). Taxifolin, luteoloside, myricetin, and quercetin experienced a highly substantial reduction with the progression of storage years (*p* < 0.01), accompanied by a notable decrease in ellagic acid (*p* < 0.05). Following 16 years of prolonged storage, their respective levels exhibited decreases of 18.5%, 50%, 75.29%, 60.55%, and 53%, respectively. Rutin and luteolin demonstrated fluctuating alterations over time but without reaching statistical significance (*p* > 0.05). Kaempferol initially underwent fluctuating changes during storage, succeeded by a rapid decline, resulting in a 69.63% reduction. Studies indicated that the aging process of Qingzhuan tea led to diminishing levels of myricetin, quercetin, and kaempferol, possibly linked to a decrease in ellagic acid, quercetin, and myricetin, and an increase in flavonoid glycosides [42]. It is hypothesized that glycosylation of flavonoid glycosides may occur during tea storage, contributing to the overall increase in total flavonoid compounds in organic green teas. This transformation of flavonol compounds into glycosylated flavonols is thought to be a complex process, with previous research speculating its association with Maillard reactions in tea or microbial catalysis during storage [33,43].

#### 3.1.4. Changes in Free Amino Acids of Organic Green Tea during Storage

Free amino acids are important biochemical components of organic green tea and contribute significantly to the flavor of the tea liquor of organic green tea. As shown in Table S2, 18 free amino acid components were detected in organic green tea samples with different storage years. The total content of free amino acids ranged from 24.68 to 53.34 mg/g, showing an initial increase followed by a decreasing trend with an increase in storage years. As shown in Figure 2, the total content was highly negatively correlated with storage year (r = −0.6985, *p* < 0.01), consistent with the changes in total amino acids. This trend might be due to the hydrolysis of some soluble proteins into free amino acids during the storage process [44], leading to an increase in free amino acids. Simultaneously, some free amino acids may undergo oxidation and degradation or combine with other chemical components to form polymers, such as high polymers resulting from the combination of free amino acids with theaflavins, thearubigins, and flavanols [12], resulting in a gradual decrease in the amino acid content during long-term storage.

The composition and content of free amino acids are essential for defining the taste of tea infusion. These free amino acids can be classified into umami, sweet, and bitter amino acids. In general, fresh amino acids include aspartic acid, glutamic acid, and theanine; sweet amino acids include proline, alanine, glycine, threonine, and serine; and bitter amino acids mainly include isoleucine, leucine, phenylalanine, tyrosine, and valine [25]. The content and proportion of umami amino acids showed an initial increase followed by a sudden decrease with an increase in storage years, highly negatively correlated with storage year (r = −0.7919, *p* < 0.01; r = −0.7101, *p* < 0.01). The content of sweet amino acids fluctuated initially and then decreased significantly with an increase in storage time (*p* < 0.05). The proportion of bitter amino acids was significantly positively correlated with storage time (r = 0.8523, *p* < 0.001) (Figure 2). The decrease in umami and sweet amino acids and the increase in bitter amino acids explain why fresh green tea is preferred by consumers.

During the storage of the organic green teas, a noteworthy correlation was observed between the content of five free amino acids (L-glutamic acid, L-proline, theanine, L-methionine, L-leucine) and the duration of storage (*p* < 0.05). Theanine, a characteristic free amino acid in tea, is the main source of fresh and crisp flavor of tea, and can work synergistically with glutamic acid and proline to enhance the fresh flavor of tea [25]. Theanine, identified as the amino acid with the highest content in organic green tea, played a pivotal role in influencing the quality changes that occurred during the storage process. Remarkably, theanine exhibited a highly significant correlation with the storage year (r = −0.7791, *p* < 0.001). As the storage years increased, the theanine content in organic green teas demonstrated an initial rise followed by a subsequent decline, reaching its peak after 5 years of storage at an impressive 32.72 mg/g. Following this peak, a gradual reduction ensued, resulting in a substantial 68.22% decrease. This observed pattern aligned with findings from various studies that have noted a consistent decline in theanine content during the storage period [15,40].

#### 3.1.5. Change in Pigments of Organic Green Tea during Storage

Tea contains a variety of tea pigments, which affect the color of dry tea and tea liquor, and their content and changes play a crucial role in tea quality. Tea pigments can be categorized into fat-soluble and water-soluble pigments. Fat-soluble pigments, primarily influencing the color of dry tea and infused leaves, encompass chlorophylls and carotenoids. Water-soluble pigments, including anthocyanins, anthocyanidins, theaflavins (TFs), thearubigins (TRs), and theabrownins (TBs), make a major contribution to the color of tea liquor, with TFs, TRs, and TBs being produced during the processing of the tea, and the content of these three pigments being higher in black tea. TRs and TFs originate from polyphenols and their derivatives through oxidative condensation. Under specific conditions, theaflavins have the capacity to undergo transformation into TRs, and subsequently, TBs result from further oxidation of both TFs and TRs [33,40]. Consequently, extended periods of storage lead to the oxidation of polyphenols in tea, resulting in the formation of TRs, TFs, and TBs. As shown in Figure 1, the TFs content in organic green teas stored over the years remained notably stable, with TRs exhibiting a marginal decrease. Conversely, the TBs content experienced a significant 46.26% increase during storage, showcasing a robust positive correlation with the duration of storage (r = 0.8765, *p* < 0.001) (Figure 2). Considering the slight increase in the content of TFs over the 5-year storage period, it is hypothesized that the oxidation of flavanols to theaflavins occurs at a faster rate than the conversion of TFs to both TRs and TBs. In the extended storage process, TRs and TFs gradually underwent oxidation, ultimately transforming into TBs.

Chlorophyll, the main component of green tea color, is one of the indicators of quality change during storage. The content of chlorophyll-a exhibited a wave-like decreasing trend during storage (*p* > 0.05), while chlorophyll-b displayed a significant negative correlation with storage year (r = −0.7321, *p* < 0.01). Chlorophyll-a is more stable than chlorophyll-b, and chlorophyll-b exhibits a more substantial loss compared to chlorophyll-a during green tea storage [32]. Carotenoids were also highly negatively correlated with storage year, showing a notable 66.18% decrease (r = −0.7279, *p* < 0.01). Research findings point to the primary cause of carotenoid reduction being the light-sensitive oxidation and degradation occurring during the storage process [38,45]. Anthocyanins, crucial antioxidants in tea [46], demonstrated a highly negative correlation with storage time, resulting in a 31.64% decrease (r = −0.9040, *p* < 0.001). This reduction has a discernible impact on the antioxidative activity of organic green tea.

### 3.2. The Multivariate Statistical Analysis Results of Functional Components of Organic Green Tea during Storage

**Figure 3:**
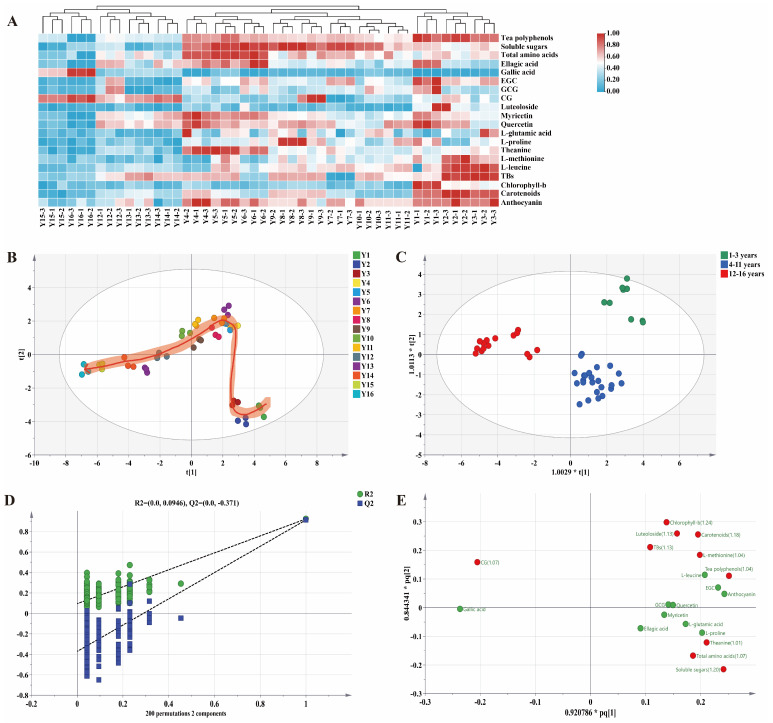
Chemometric analysis of compounds during long-term storage of organic green tea. (**A**) Hierarchical clustering heat map of 20 differential compounds. (**B**) PCA plots of organic green tea compounds in different storage years. (**C**) OPLS-DA scoring plot of compounds during storage of organic green tea. (**D**) Cross-validation of the OPLS-DA model. (**E**) Loading plot. Red dots indicate compounds with VIP values greater than 1 (VIP > 1).

Exploring the correlation between the content of the functional components of organic green tea and storage year through correlation analysis can provide a deeper understanding of the changes in functional components of organic green tea during storage. Under the condition of |r| > 0.5 and *p* < 0.05 (r is the correlation coefficient), this study utilized Pearson correlation analysis to unveil the intricate relationship between functional components in organic green tea and storage year, pinpointing 20 functional compounds that demonstrated significant correlations with storage years (Figure 3A, Table S3). Notably, 17 of these functional compounds (tea polyphenols, soluble sugars, total amino acids, anthocyanin, luteoloside, ellagic acid, theanine, carotenoids, EGC, GCG, myricetin, chlorophyll-b, L-methionine, quercetin, L-proline, L-leucine, and L-glutamic acid) displayed a significant negative correlation with storage time (r < −0.5), signifying a gradual decrease in their content throughout the storage process. This decline had implications for the taste and appearance of organic green tea. Conversely, three compounds (TBs, gallic acid, CG) exhibited a significant positive correlation with storage year (r > 0.5), showcasing a marked increase in their content during the storage period. This finding provides valuable insights into the mechanisms underlying the browning of organic green tea, as well as the degradation and transformation of catechins throughout the storage duration.

The 20 functional components that show significant correlations with storage year were analyzed through both PCA and HCA. PCA results (Figure 3B) indicated that sample data from the same storage year clustered closely together, demonstrating good repeatability and high reliability of the organic green tea samples. Samples with different storage years could be clearly distinguished, and the functional components of organic green tea changed along the PC1 direction over the storage years, suggesting significant alterations in the functional composition of organic green tea during the storage period.

Based on HCA (Figure 3A), the organic green tea samples were effectively categorized into three distinct clusters: one cluster encompassing storage periods from 1 to 3 years, another for storage periods spanning 4 to 11 years, and the third for storage periods ranging from 12 to 16 years. Employing the 20 functionally correlated components as dependent variables and storage year as the independent variable, OPLS-DA was conducted. As shown in the Figure 3C, the OPLS-DA model exhibited a notable ability to discriminate between organic green teas with short- and long-term storage periods (R^2^x = 0.731, R^2^y = 0.950, Q^2^ = 0.941). The cross-validation through 200 permutation tests revealed that the point of intersection with the vertical axis below 0 in Q^2^ indicated no overfitting in the model (R^2^ = 0.0946, Q^2^ = −0.371), affirming its efficacy in accurately distinguishing organic green teas with varying storage years (Figure 3D).

Additionally, to delve deeper into the analysis of the contribution of functional components in distinguishing organic green teas of varying storage years, the variable importance in the projection (VIP) value for each compound can be derived through OPLS-DA. The VIP value serves to characterize the distinct contribution of compounds in different samples. By evaluating variable importance in the OPLS-DA model and combining ANOVA and Kruskal–Wallis tests, 10 key compounds, namely, chlorophyll-b, soluble sugars, carotenoids, luteoloside, TBs, total amino acids, CG, tea polyphenols, L-methionine, and theanine, were identified based on the criteria of VIP ≥ 1 and *p* < 0.05, effectively distinguishing organic green teas with different storage years (Figure 3E).

### 3.3. The Taste Analysis of Organic Green Tea during Storage Based on Electronic Tongue

**Figure 4:**
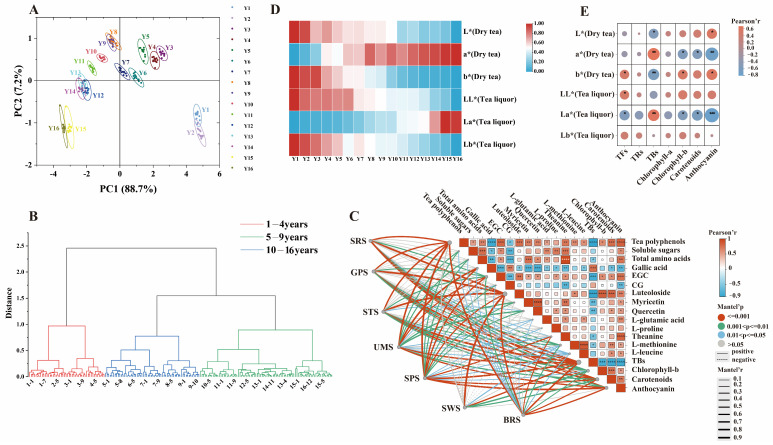
Taste and color changes in organic green tea during long-term storage based on electronic tongue and color differences. (**A**) PCA plots of taste change in organic green tea. (**B**) Hierarchical cluster analysis of organic green tea during long-term storage based on electronic tongue. (**C**) Correlation analysis between taste characteristics and important functional components. (**D**) Changes in color of dry tea and tea liquor of organic green tea. (**E**) Relationships between color difference values and tea pigments. **** *p* < 0.0001, *** *p* < 0.001, ** *p* < 0.01, * *p* < 0.05, Significant correlation.

The taste evaluation of organic green tea stored year by year was performed by the electronic tongue technique. As the storage years increased, the response values of various sensors exhibited a notable decrease. Except for SPS and STS, the response values from the five sensors for organic green tea samples with varying storage years displayed significant dispersion, underscoring the high sensitivity of these sensors to specific substances within the samples (Figure S2). Analyzing Figure S2, it becomes evident that aside from the tea samples stored for 1 and 2 years, the differences in sweetness among samples stored for other durations are relatively minor. This trend corresponds with observed variations in the sweetness of oolong tea during storage [9]. The freshness of tea samples stored for 15 and 16 years notably diminished compared to those stored for other durations (*p* < 0.05), aligning with changes in the content of free amino acids, particularly theanine. Acidic compounds in tea infusions dissociated, yielding hydrogen ions that imparted acidity to the tea [8]. This acidity tended to decrease during the storage of organic green tea, consistent with the trend in oolong tea at different storage years [9] but contrasting with large-leaf black tea [8] at varying storage years. A moderate bitterness contributes to the mellow quality of tea [8]. This bitterness in organic green tea decreases with an increase in storage years, echoing trends observed in the bitterness of Dancong tea at different storage periods [9]. As shown in Figure 4A, PCA of the electronic tongue pattern for different storage years of organic green tea revealed that PC1 (88.7%) and PC2 (7.2%) contributed to a total variance of 95.9%, reflecting comprehensive information about the samples. Different storage years of organic green tea were clearly distinguishable, with the taste gradually changing with increased storage years, primarily differentiated by PC1. The distinction between teas stored for 1 and 2 years, 15 and 16 years, and those stored for other durations primarily depended on PC2, indicating the efficacy of the electronic tongue in differentiating taste characteristics at various storage years. HCA of the electronic tongue response values classified organic green teas with different storage times into three clusters: stored for 1–4 years, 5–9 years, and 10–16 years (Figure 4B). Storage time significantly influenced the taste of tea infusions, with teas stored for 1–4 years exhibiting similar taste qualities and those stored for 5–9 and 10–16 years clustering together, suggesting a high similarity in taste characteristics within these respective groups.

To comprehend the correlation between taste quality and the primary functional components of organic green tea, various taste characteristics obtained from the electronic tongue were subjected to Pearson’s correlation analysis with the major functional components (Table S4). Under the condition of |r|> 0.6, *p* < 0.05, 18 functional components were selected to significantly contribute to the taste quality during the storage of organic green tea, including total amino acids, soluble sugars, tea polyphenols, gallic acid, CG, EGC, quercetin, myricetin, luteoloside, TBs, chlorophyll-b, carotenoids, anthocyanin, L-methionine, L-proline, L-leucine, theanine, and L-glutamic acid. The relationships between different electronic tongue taste-test features and major functional components is shown in Figure 4C. Specifically, the flavor profile of organic green tea was associated with total amino acids, soluble sugars, tea polyphenols, four phenolic compounds (quercetin, myricetin, luteoloside, EGC), five amino acids (L-methionine, L proline, L-leucine, theanine, L-glutamic acid), and three pigment components (chlorophyll-b, carotenoids, anthocyanin), showing a significant positive correlation (r > 0.6). To the contrary, the changes in gallic acid, CG, and TRs showed a significant negative correlation (r < −0.6) with the flavor profile of organic green tea. Integrating both chemical quantitative analysis and stringent criteria (VIP ≥ 1, |r|> 0.6, and *p* < 0.05), we identified nine functional components as key compounds influencing the taste changes of organic green tea during storage, including total amino acids, soluble sugars, tea polyphenols, L-methionine, theanine, luteoloside, chlorophyll-b, carotenoids, and CG.

### 3.4. The Chromatic Analysis of Organic Green Tea during Storage Based on Colorimeter

Tea color is a crucial evaluation criterion for assessing the sensory quality of tea. Figure 4D illustrates the influence of storage year on the color attributes of both the dry tea and tea liquor of organic green tea. As the storage years increased, the L* of both the dry tea and tea liquor consistently decreased, corresponding to the gradual darkening of the appearance of organic green dry tea and the color of the tea liquor. The a* of the dry tea consistently exhibited positive values, showcasing an increasing trend with storage year and eventually registering a 66.37% increase. In contrast, the b* of the dry tea decreased by 59.11% by the end of the storage period. These changes signify a reduction in yellow-green color and an enhancement of red color in the dry tea. Meanwhile, the a* of the tea liquor consistently demonstrated negative values, indicating a decrease in green attributes. The a* of the tea liquor of organic green tea, across different storage years, remained consistently negative, while the b* consistently registered positive values, signifying a yellow-green color in the tea liquor. With increasing storage years, the a* value in the tea liquor experienced a 75% increase, indicating a decrease in the green attribute. Simultaneously, the overall trend in b* descended, suggesting a decline in the yellow attribute. These observations imply a gradual reduction in the yellow-green color in the tea liquor of organic green tea. It can be inferred that the variations in L*, a*, and b* values for both the dry tea and the tea liquor of organic green tea follow a consistent trend. Teas stored for longer durations exhibit a darker and deeper color, emphasizing intensified red attributes and a diminished yellow-green attribute.

ΔE represents the total color difference of organic green tea, combining the color difference change attributes of the ΔL*, Δa*, and Δb*. The larger the ΔE value, the larger the color difference; when ΔE is between 0 and 1, the color difference is indistinguishable to the naked eye, when ΔE is between 1 and 2, the color difference is slightly noticeable, when 3.5 < ΔE < 5, the color difference is visible to the naked eye, and when ΔE > 5, the color difference is larger, implying two different colors [47]. The color differences (ΔE) in both the dry tea and tea liquor of annually stored organic green teas are presented in Table 1. As the storage years progressed, the appearance of ΔE of organic green tea gradually rose. For instance, the ΔE between the organic tea stored for one year and three years was 6.45, indicating a noticeable color distinction. Particularly noteworthy was the ΔE between organic green tea stored for 16 years and for 1 year, which escalated to 21.93, underscoring a significant visual contrast. Moreover, during the 2nd and 3rd years, as well as between the 10th and 11th years of storage, the color differences exhibited notable gaps, all exceeding 4. This suggested that after 3 and 11 years of storage, discernible shifts in the visual color profile of organic green tea had already taken place. Correspondingly, the ΔE in the tea liquor of organic green tea progressively increased with each storage year. The ΔE between the tea liquor of the year 1 and year 16 stored organic green tea reached 17.12, with pronounced color variations observed between the 6th and 7th storage years. This apparent transformation signals a substantial modification in the color quality of the tea liquor during this period.

In order to measure the degree of correlation between the color change of organic green tea and tea pigments, Pearson correlation analysis was performed on the L*, a*, and b* values of dry tea and tea liquor tea pigments (Table S5). Based on |r| > 0.5, and *p* < 0.05, Figure 4E elucidates significant correlations between the L* and b* values of dry tea and the presence of anthocyanin and TBs (*p* < 0.05). Notably, the L* and b* of dry tea exhibited a positive correlation (r > 0.5) with anthocyanins and a negative correlation (r < −0.5) with TBs. The a* values of both dry tea and tea liquor were significantly associated with chlorophyll-b, carotenoids, anthocyanin, and TBs (*p* < 0.05). Specifically, a* correlated positively with TBs (r > 0.5) and negatively with chlorophyll-b, carotenoids, and anthocyanins (r < −0.5). Furthermore, there were noteworthy differences (*p* < 0.05) in the L* and a* of tea liquor and b* of dry tea concerning TFs. The L* of tea liquor and b* of dry tea demonstrated a positive correlation (r > 0.5) with TFs, while the a* of tea liquor exhibited a negative correlation with TFs (r < −0.5). As anthocyanins, carotenoids, and chlorophyll-b decreased over prolonged storage, TBs showed an increasing trend. Consequently, the L* and b* values of dry tea and tea soup decreased, while a* values increased, indicating a darkening of color and a reduction in green and yellow tones. This indicates that the decline in anthocyanins, carotenoids, and chlorophyll-b, coupled with the rise in TBs, adversely affects the color quality of both the dry tea and tea liquor of annually stored organic green tea.

### 3.5. The Classification Predication Analysis of the Storage Years of Organic Green Tea Based on BPNN

A BPNN was utilized for the classification of organic green tea with different storage years. The dataset comprised electronic tongue response values (sample size of 192), color difference values of dry tea and tea liquor (sample size of 144), and the content of 10 selected functional components (VIP ≥ 1, *p* < 0.05, sample size of 48) determined by the OPLS-DA model. Due to the ability of electronic tongue technology to accurately and comprehensively detect the taste variations in organic green tea during storage, the classification prediction based on electronic tongue signals successfully categorized the teas into three groups: 1–4 years, 5–9 years, and 10–16 years.

**Figure 5:**
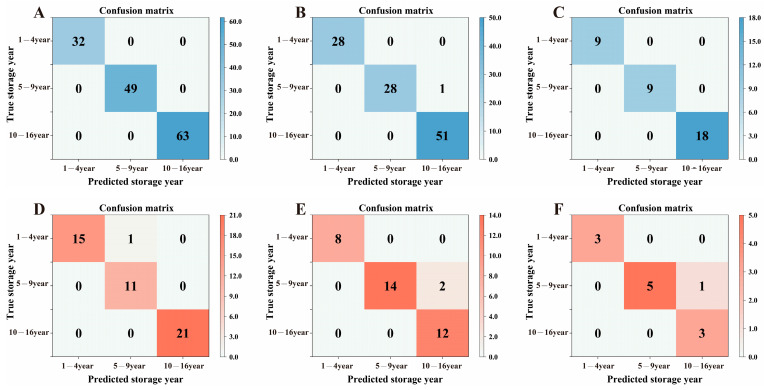
Confusion matrix for the BPNN discriminant model. (**A**) Training set based on electronic tongue. (**B**) Training set based on 10 compounds. (**C**) Training set based on color difference value. (**D**) Test set based on electronic tongue. (**E**) Test set based on 10 compounds. (**F**) Test set based on color difference value.

In the established BPNN model, the accuracy of the test set for the three types of data varied significantly, with the model based on the 10 components achieving an accuracy of 91.67%, the model based on the color difference data achieving an accuracy of 94.44%, and the model based on the electronic tongue signal achieving the highest accuracy of 97.92%. The confusion matrix (Figure 5) illustrates the classification of the three models. It was noteworthy that in the confusion matrix of the test set for models based on the 10 components and color difference data, there was a consistent phenomenon wherein samples with storage years falling within years 5–9 were misclassified as falling within years 10–16. Specifically, in the model with color difference data, two samples with 5–9 years of storage were misclassified as having been stored for 10–16 years, while in the model based on the 10 components, one sample with 5–9 years of storage was misclassified as having been stored for 10–16 years. This indicated a certain similarity between the two models in discriminating the storage of organic green tea, and it was speculated that this might be due to the influence of color-contributing components. The color alteration in organic green tea during long-term storage was primarily attributed to colored substances such as flavonols, chlorophylls, carotenoids, TFs, TBs, and TFs. The dataset was established based on 10 key compounds (VIP ≥ 1), including significant pigment substances (chlorophyll-b, carotenoids, TBs) that impacted the color change in both dry tea and the tea liquor of organic green tea. These three key pigments, with larger VIP values (Figure 3E), significantly influenced the color changes in both dry tea and tea liquor, making a considerable contribution to the storage discrimination of organic green tea.

The mean square error (MSE) and the correlation coefficient (R) are important parameters for evaluating the performance of neural network models. The optimal hidden-layer neurons for the BPNN model can be determined by the minimum MSE and maximum R [48]. According to Table 2, in the established BPNN model, the optimal number of hidden-layer neurons for the model with the dataset of electronic tongue signals was eight, MSE = 3.4907 × 10^−7^, R = 1, and the network structure was 7-8-3. The optimal number of hidden-layer neurons for the model with a dataset of 10 functional components was nine, MSE = 0.0031345, R = 0.99509, and the network structure was 10-9-3. The optimal number of hidden-layer neurons for the model with the dataset of color difference values was five, MSE = 0.000813, R = 0.99876, and the network structure was 6-5-3. Among the three models, the one based on electronic tongue signals exhibited the smallest MSE and the largest correlation coefficient. Combined with the parametric performance of the models (Table S6), the results indicated that the classification and prediction capability of the BPNN model based on electronic tongue signals surpassed the models based on color difference values and 10 functional components (VIP ≥ 1), which also verified the reliability of the cluster analysis of the long-term storage of organic green tea based on electronic tongue signals.

### 3.6. Comparative Analysis of the In Vitro Antioxidant Capacity among Organic Green Teas during Storage

**Figure 6:**
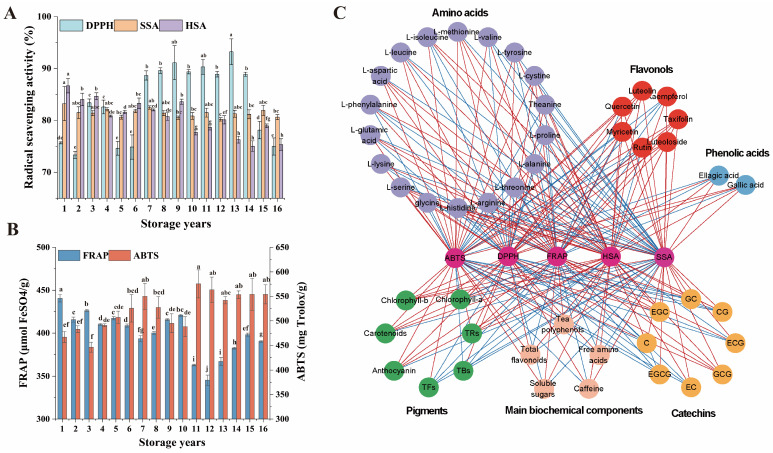
Antioxidant capacity analysis of organic green tea. (**A**) The in vitro antioxidant activity of organic green teas during long-term storage determined by DPPH, HSA and SSA. (**B**) The in vitro antioxidant activity of organic green teas during long-term storage determined by FRAP and ABTS. Different letters represent significance at the level of *p* < 0.05. (**C**) Correlation network of in vitro antioxidant activity and 47 compounds. Line color represents the positive (red) or negative (blue) correlation.

Tea is recognized as a valuable dietary source of natural antioxidants, primarily derived from its functional components [4]. The antioxidative capacity of organic green tea samples during long-term storage was evaluated through DPPH, HSA, SSA, ABTS, and FRAP assays. As shown in Figure 6A,B, SSA, HSA, and FRAP exhibited the highest values in organic green tea samples stored for one year, gradually decreasing with prolonged storage; in particular, HSA and FRAP significantly decreased (*p* < 0.01), while ABTS displayed an opposing trend (*p* < 0.01). DPPH demonstrated a parabolic pattern, reaching its peak in organic green teas stored for 7–14 years. These findings suggest that short-term storage of organic green tea confers notable advantages in inhibiting superoxide anion free radicals, reducing iron ions, and suppressing hydroxyl radicals. However, the antioxidative capabilities against lipid- and water-soluble free radicals were comparatively weaker. This aligns with previous research indicating a significant enhancement in DPPH capacity during the aging process of white tea [7].

Pearson correlation analysis between the antioxidative activity and functional components of organic green tea revealed significant correlations (Figure 6C, Table S7). Notably, DPPH showed a significant positive correlation with L-serine (*p* < 0.05) and a significant negative correlation with luteoloside (*p* < 0.05). ABTS showed significant positive correlation with L-serine, L-alanine, and TBs (*p* < 0.05), and significant negative correlation (*p* < 0.05) with EGC, luteoloside, L-glutamic acid, L-methionine, tea polyphenols, carotenoids, chlorophyll-b and anthocyanin (*p* < 0.05). FRAP was significantly positively correlated with luteoloside, L-glutamic acid, L-methionine, tea polyphenols, carotenoids, chlorophyll-b, and anthocyanin (*p* < 0.05), and significantly negatively correlated with L-serine and TBs (*p* < 0.05). SSA was significantly positively correlated with taxfolin, luteoloside, luteololin, chlorophyll-b, EGC, EC, and C (*p* < 0.05), and significantly negatively correlated with TBs (*p* < 0.05). HSA was significantly and positively correlated with EGC, GC, GCG, taxifolin, luteoloside, L-proline, theanine, L-methionine, tea polyphenols, soluble sugars, carotenoids, chlorophyll-b, and anthocyanin (*p* < 0.05), and highly significantly negatively correlated with gallic acid and TBs (*p* < 0.01). Polyphenols are recognized as crucial bioactive components in tea, including catechins, flavones, flavonols, anthocyanins, and phenolic acids. These compounds are essential active substances beneficial to human health [4]. Apart from polyphenols, amino acids, particularly theanine, have demonstrated antioxidant properties [49]. The potent capability of short-term-stored organic green tea to inhibit superoxide anion radicals, hydroxyl radicals, and reduce iron ions was attributed to the elevated content of taxifolin, luteoloside, L-methionine, tea polyphenols, carotenoids, chlorophyll-b and anthocyanin. The substantial reduction of these eight compounds during the long-term storage of organic green tea contributed to the observed changes. On the other hand, long-term-stored organic green tea demonstrated strong antioxidant abilities in clearing lipid- and water-soluble free radicals. The possible reason for this may be the high content of L-serine and TBs, etc., which were significantly elevated during long-term storage. Previous studies have indicated the antioxidant potential of TBs [50]. In summary, the antioxidative activity of organic green tea is closely related to the changes in the content of phenolic compounds, such as tea polyphenols, luteoloside, carotenoids, chlorophyll-b, anthocyanin, and TBs, etc. Additionally, changes in some free amino acids have a certain impact on the antioxidative activity of organic green tea.

## 4. Conclusions

This study represents the first comprehensive analysis of the major chemical compounds, taste, color, and antioxidative performance of organic green tea during long-term storage. We measured 47 main functional components, revealing significant negative correlations between storage year and tea polyphenols, total amino acids, soluble sugars, two phenolic acids, four flavonols, three pigments, fresh amino acids, and sweet amino acids (*p* <0.05). The multivariate statistical analysis revealed that 10 functional components that effectively differentiated organic green tea during storage could be selected. Electronic tongue technology successfully classified organic green tea into three categories: 1–4 years, 5–9 years, and 10–16 years. Colorimeter analysis unveiled that the tea color darkened with prolonged storage, red attributes intensified, and yellow-green attributes diminished. Subsequently, BPNN classification analysis demonstrated that the classification model accuracy based on the electronic tongue surpassed that of the model based on color difference values and 10 functional components, with VIP > 1. Comprehensive antioxidant activity and functional compounds data showed that organic green tea during short-term storage had strong superoxide anion radical and hydroxyl radical inhibition and ferric ion reduction capacities. The organic green tea during long-term storage had stronger antioxidant activity in scavenging fat-soluble and water-soluble free radicals. This study provides a theoretical reference for discriminating and evaluating the quality of green tea during storage, contributing to the standardization of the tea market and the protection of consumer interests.

## Figures and Tables

**Table 1:** ΔE difference of dry tea and tea liquor of organic green teas during long-term storage.

	Dry Tea															
	Y1	Y2	Y3	Y4	Y5	Y6	Y7	Y8	Y9	Y10	Y11	Y12	Y13	Y14	Y15	Y16
Y1	0.00															
Y2	1.68	0.00														
Y3	6.45	4.99	0.00													
Y4	6.13	4.47	3.04	0.00												
Y5	8.39	6.72	3.76	2.32	0.00											
Y6	9.84	8.16	5.03	3.72	1.48	0.00										
Y7	9.70	8.02	4.97	3.58	1.36	0.17	0.00									
Y8	10.92	9.25	6.24	4.80	2.68	1.24	1.34	0.00								
Y9	11.09	9.42	6.53	5.00	2.89	1.49	1.57	0.46	0.00							
Y10	12.88	11.24	8.59	6.90	4.92	3.56	3.63	2.40	2.07	0.00						
Y11	16.69	15.02	11.48	10.60	8.34	6.88	7.02	5.86	5.67	4.30	0.00					
Y12	16.60	14.94	11.60	10.52	8.32	6.85	6.98	5.76	5.54	3.95	0.76	0.00				
Y13	18.20	16.53	12.96	12.11	9.86	8.39	8.53	7.35	7.16	5.68	1.53	1.74	0.00			
Y14	18.10	16.43	12.91	12.01	9.77	8.30	8.44	7.24	7.05	5.54	1.45	1.59	0.21	0.00		
Y15	18.88	17.21	13.59	12.77	10.52	9.06	9.20	8.01	7.84	6.36	2.21	2.42	0.69	0.84	0.00	
Y16	21.93	20.26	16.29	15.85	13.55	12.13	12.28	11.17	11.04	9.72	5.45	5.81	4.07	4.24	3.42	0.00
	**Tea Liquor**															
Y1	0															
Y2	2.41	0.00														
Y3	3.28	0.92	0.00													
Y4	3.54	1.44	0.73	0.00												
Y5	4.37	2.24	1.38	0.84	0.00											
Y6	4.92	3.09	2.34	1.66	1.04	0.00										
Y7	7.72	5.35	4.45	4.27	3.52	3.63	0.00									
Y8	8.13	5.78	4.87	4.65	3.88	3.89	0.49	0.00								
Y9	8.63	6.29	5.37	5.14	4.36	4.29	1.01	0.52	0.00							
Y10	9.37	7.00	6.10	5.91	5.14	5.11	1.66	1.26	0.82	0.00						
Y11	9.51	7.17	6.26	6.03	5.25	5.15	1.87	1.41	0.91	0.43	0.00					
Y12	9.77	7.44	6.53	6.26	5.46	5.29	2.19	1.70	1.18	0.81	0.44	0.00				
Y13	10.65	8.33	7.41	7.15	6.34	6.13	3.05	2.57	2.06	1.50	1.21	0.89	0.00			
Y14	13.02	10.67	9.77	9.53	8.74	8.54	5.36	4.91	4.41	3.71	3.51	3.28	2.41	0.00		
Y15	14.11	11.77	10.87	10.64	9.85	9.64	6.49	6.04	5.54	4.84	4.64	4.40	3.55	1.18	0.00	
Y16	17.12	14.75	13.86	13.65	12.86	12.67	9.43	9.01	8.51	7.77	7.62	7.40	6.54	4.13	3.08	0.00

**Table 2:** Effect of the number of hidden-layer neurons on the BPNN model for discriminating the storage year of organic green tea.

Date Sources	Hidden-Layer Neurons	MSE	R
Electronic tongue	3	1.4339 × 10^−6^	1
	4	3.7988 × 10^−6^	0.99999
	5	1.007 × 10^−4^	0.99984
	6	4.0372 × 10^−7^	1
	7	4.5154 × 10^−4^	0.99928
	8	3.4907 × 10^−7^	1
	9	1.7369 × 10^−6^	1
	10	5.0162 × 10^−5^	0.99992
	11	1.4991 × 10^−5^	0.99998
	12	9.9914 × 10^−5^	0.99984
10 compounds (VIP ≥ 1)	4	0.11556	0.81912
	5	0.049652	0.92228
	6	0.0087612	0.98629
	7	0.014276	0.97766
	8	0.033338	0.94782
	9	0.0031345	0.99509
	10	0.010991	0.98280
	11	0.030782	0.95182
	12	0.0039172	0.99387
	13	0.0053279	0.99166
Chromatism	3	0.032164	0.95102
	4	0.049487	0.92464
	5	0.000813	0.99876
	6	0.001205	0.99816
	7	0.001968	0.99700
	8	0.025203	0.96162
	9	0.003561	0.99799
	10	0.001317	0.99799
	11	0.040915	0.93769
	12	0.001798	0.99726

## Data Availability

The original contributions presented in the study are included in the article and Supplementary Materials, further inquiries can be directed to the corresponding author.

## Supplementary Materials

The following supporting information can be downloaded at: https://www.mdpi.com/article/10.3390/foods13050753/s1, Figure S1: Schematic diagram of the structure of BPNN model; Figure S2: Radar plots based on e-tongue response values of organic green teas of different storage years; Table S1: Detailed information of organic green tea with different storage years; Table S2: Content of functional components of organic green tea of different storage years; Table S3: The correlation coefficient between 20 functional compounds in organic tea and storage year; Table S4: The correlation coefficient between 18 functional compounds in organic tea and electronic tongue signals; Table S5: The correlation coefficient between the color difference values of dry tea and tea liquor and tea pigments; Table S6: BPNN classification results and model performance metrics based on electronic tongue, chromatism, and 10 compounds (VIP ≥ 1); Table S7: The correlation coefficient between the antioxidative activity and functional components of organic tea.
