# Supplementary material for: The Flavor Characteristics, Antioxidant Capability, and Storage Year Discrimination Based on Backpropagation Neural Network of Organic Green Tea (Camellia sinensis) during Long-Term Storage
Source: Foods. 2024 Feb 29;13(5):753. doi: 10.3390/foods13050753 (PMC10930645; doi:10.3390/foods13050753)
Supplement: Supplementary file 1 [file foods-13-00753-s001.zip › foods-2885530-supplementary.pdf]

Supplementary Materials for

The Flavor Characteristics, Antioxidant Capability, and Storage Year Discrimination Based on Backpropagation Neural Network of Organic Green Tea (*Camellia sinensis*) during Long-Term Storage

In vitro antioxidant capacity evaluation by five various assays

FRAP assay

Weigh 1g of organic green tea sample and add 4 mL of physiological saline in a 1:4 ratio of weight (g) to volume (mL). The sample was mechanically homogenized under ice water bath conditions, and then centrifuged at 4°C and 12000 r/min for 5 min before collecting the supernatant. Use an enzyme-linked immunosorbent assay analyzer to measure at 593 nm. The FRAP values of the organic green tea samples were calculated using $FeSO_4$ as the standard, and the calibration curve was established as follows:

$$Y_1=3.0713X_1+0.276 \quad (R^2=0.9962) \tag{1}$$

X_1 is the concentration of $FeSO_4$, mmol/L; Y_1 represents OD_{593}.

ABTS assay

Accurately weigh 0.1 g of organic green tea sample and add 0.9 mL of physiological saline in a ratio of weight (g) to volume (mL)=1:9. Mechanical homogenization under ice water bath conditions, centrifugation at 12000 r/min for 5 min, and then take the supernatant. Measure at 405 nm wavelength using an enzyme-linked immunosorbent assay (ELISA) analyzer. The ABTS value of organic green tea samples was calculated using Trolox as the standard, and a calibration curve was established as follows:

$$Y_2=-1.1215X_2+1.1262 \quad (R^2=0.9957) \tag{2}$$

X_2 is the concentration of Trolox, mmol/L; Y_2 represents OD_{405}.

DPPH assay

Weigh 0.1g of organic green tea sample, add 1 mL of 80% methanol solution, and homogenize in an ice water bath. Centrifuge at 12000 r/min for 10 min and then take the supernatant. Measure the sample at 517 nm using a UV visible spectrophotometer. The DPPH radical scavenging rate based on equation (3):

$$\text{DPPH radical scavenging rate (\%)}=[A_{blank}-(A_{test}-A_{control})]/A_{blank}*100\% \tag{3}$$

HSA assay

Take 0.1 g of organic green tea sample, add 5 mL of distilled water and extract in

boiling water bath for 10 min, centrifuge at 12000 r/min for 10 min and then take the supernatant and dilute 10 times. It was measured at 550 nm using UV-visible spectrophotometer. Calculate the rate of HSA according to equation (4):

$$\text{Rate of HSA}(\%)=[A_{blank}-(A_{test}-A_{control})]/A_{blank}*100\% \quad (4)$$

SSA assay

Weigh 0.1 g of organic green tea sample, add 5 mL of distilled water in a boiling water bath for 10 min, centrifuge at 12000 r/min for 10min and then take the supernatant and dilute 10 times. It was measured at 550 nm using UV-visible spectrophotometer. Calculate the rate of SSA according to equation (5):

$$\text{Rate of SSA}(\%) = (A_{control}-A_{test})/(A_{control}-A_{blank})*100\% \quad (5)$$

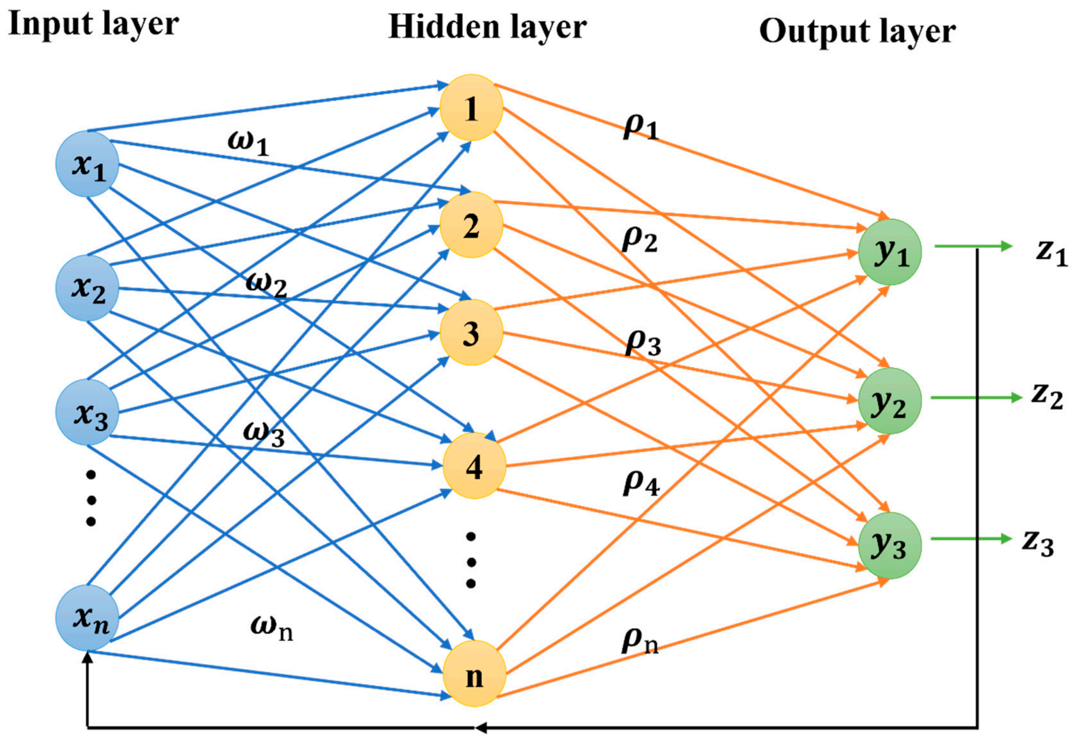

Figure S1. Schematic diagram of the structure of the BPNN model.

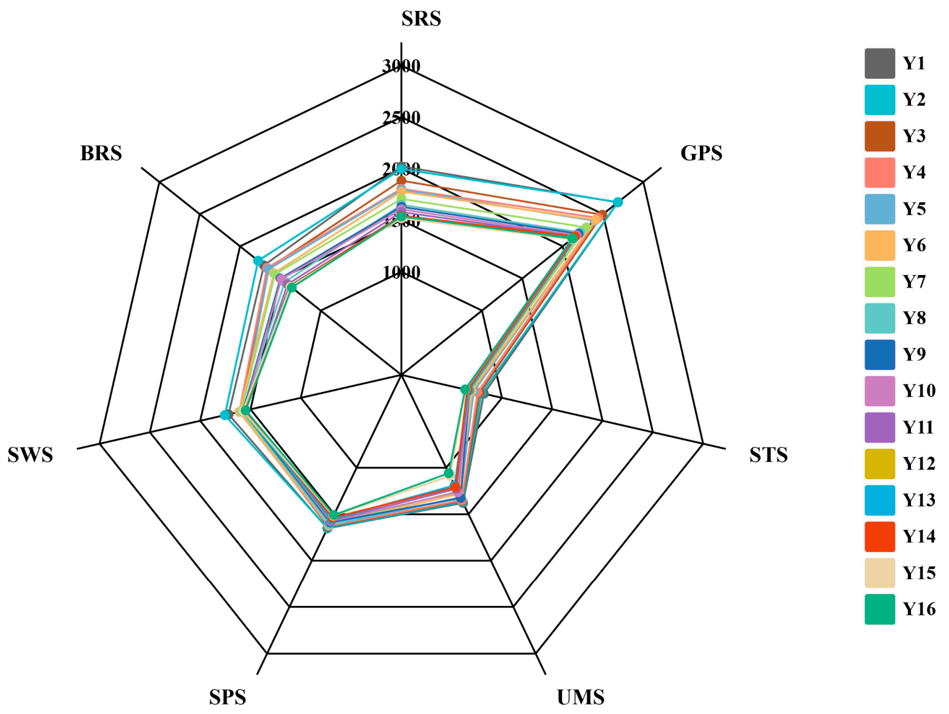

Figure S2. Radar plots based on electronic tongue response values of organic green teas of different storage years.

Table S1. Detailed information of organic green tea with different storage years.

Samples	Production year	Storage year	Standard for picking fresh leaves	Production area
Y1	2021	1		
Y2	2020	2		
Y3	2019	3		
Y4	2018	4		
Y5	2017	5		
Y6	2016	6		
Y7	2015	7		
Y8	2014	8		
Y9	2013	9	One bud and two leaves	Lishui City, Zhejiang Province, China
Y10	2012	10		
Y11	2011	11		
Y12	2010	12		
Y13	2009	13		
Y14	2008	14		
Y15	2007	15		
Y16	2006	16		

Table S2. Content of functional compounds in organic green tea samples with different storage years.

Compounds (mg/g)	Storage years															
	1	2	3	4	5	6	7	8	9	10	11	12	13	14	15	16
Tea polyphenols(%)	14.68±0.4	14.73±0.14	14.23±0.06	13.91±0.13	14.12±0.35	13.58±0.12	13.48±0.06	13.17±0.10	13.30±0.10	13.04±0.26	12.96±0.11	12.69±0.11	12.47±0.05	12.23±0.06	12.28±0.05	10.55±0.05
Total flavonoids	18.61±0.09	25.71±0.09	23.65±0.06	19.12±0.06	18.04±0.13	13.57±0.06	21.56±0.09	18.47±0.11	18.53±0.09	16.47±0.06	19.16±0.06	23.55±0.09	17.52±0.09	14.54±0.06	21.69±0.13	21.37±0.13
Soluble sugars	38.08±0.43	34.45±0.43	37.24±1.54	42.92±0.70	46.18±0.43	46.46±0.70	45.72±0.16	47.11±0.16	43.95±1.06	43.58±0.28	37.90±1.59	32.77±0.43	25.51±0.70	28.21±0.28	24.39±0.85	29.89±0.28
Total amino acids	41.06±3.50	41.41±3.54	43.13±1.08	52.13±0.22	54.12±0.32	53.52±1.46	45.96±0.67	42.23±2.87	42.90±4.75	42.08±1.56	42.23±2.87	41.00±1.88	40.90±3.74	36.16±1.78	32.22±0.06	30.07±5.86
Caffeine	29.49±0.91	30.54±0.68	33.79±1.92	31.16±0.73	31.06±0.80	29.69±1.27	36.42±0.52	33.13±0.65	34.98±1.01	33.96±1.76	34.38±0.56	31.24±2.11	31.16±0.65	31.43±0.31	32.70±2.21	35.72±1.07
GA	0.01±0.00	0.02±0.00	0.02±0.00	0.02±0.00	0.06±0.01	0.02 ±0.00	0.03±0.00	0.04±0.00	0.04±0.00	0.06±0.00	0.04±0.00	0.07±0.01	0.09±0.00	0.09±0.00	0.17±0.00	0.26±0.01
GC	0.04±0.00	0.02±0.00	0.02±0.00	0.02±0.00	0.02±0.00	0.03±0.00	0.04±0.00	0.03±0.00	0.02±0.00	0.02±0.00	0.02±0.00	0.04±0.01	0.01±0.00	0.01±0.00	0.02±0.00	0.02±0.00
EGC	1.20±0.08	0.92±0.05	0.96±0.08	0.88±0.05	0.78±0.09	0.96±.06	1.01±0.04	0.79±0.02	0.73±0.01	0.76±0.13	0.71±0.05	0.89±0.18	0.49±0.01	0.42±0.02	0.48±0.03	0.45±0.01
C	0.05±0.00	0.02±0.00	0.02±0.00	0.03±0.00	0.02±0.00	0.03±0.00	0.08±0.00	0.05±0.01	0.03±0.01	0.02±0.01	0.03±0.01	0.03±0.01	0.01±0.00	0.01±0.00	0.03±0.00	0.03±0.00
EGCG	66.27±9.42	33.79±4.76	22.22±3.80	47.19±1.53	12.36±2.35	67.01±7.57	41.11±4.16	23.57±4.07	70.68±3.33	76.25±18.24	54.90±5.53	59.83±7.33	31.20±5.43	22.82±1.64	52.35±2.34	42.25±3.38
EC	0.52±0.02	0.31±0.02	0.32 ±0.02	0.29±0.02	0.27±0.03	0.35±0.14	1.03±0.04	0.48±0.12	0.36±0.01	0.23±0.04	0.27±0.02	0.28±0.04	0.15±0.01	0.16±0.01	0.18±0.01	0.14±0.00
GCG	0.78±0.15	0.37±0.05	0.35±0.04	0.42±0.02	0.26±0.03	0.56±0.09	0.45±0.03	0.34±0.05	0.42±0.01	0.55±0.15	0.47±0.01	0.63±0.20	0.11±0.01	0.21±0.01	0.19±0.04	0.19±0.01
ECG	4.83±0.73	2.09±0.34	1.08±0.22	2.39±0.08	1.25±0.28	1.99±0.27	4.52±0.67	1.05±0.35	4.28±0.19	4.52±1.14	5.19±0.33	5.76±1.43	3.31±0.42	1.44±0.16	3.95±0.48	3.45±0.33
CG	0.01±0.00	0.01±0.00	0.01±0.00	0.01±0.00	0.01±0.00	0.01±0.00	ND	0.01±0.00	0.02±0.01	0.01±0.00	0.01±0.00	0.01±0.00	0.01±0.00	0.02 ±0.00	0.02 ±0.00	0.02±0.00
Taxifolin	2.81±0.03	2.57±0.14	2.62±0.43	2.88±0.49	2.72±0.68	2.71±0.89	2.91±0.74	2.97±0.60	2.22±0.12	2.17±0.30	2.24±0.03	2.12±0.08	2.26±0.09	2.37±0.29	2.42±0.13	2.29±0.22
Luteoloside	0.2±0.06	0.20±0.06	0.16±0.01	0.13±0.01	0.12±0.03	0.14±0.04	0.12±0.02	0.11±0.01	0.11±0.01	0.10±0.00	0.09±0.00	0.11±0.01	0.10±0.01	0.09±0.01	0.09±0.01	0.10±0.00
Rutin	0.50±0.04	0.62±0.07	0.53±0.07	0.53±0.03	0.59±0.16	0.56±0.02	0.73±0.08	0.64±0.04	0.62±0.02	0.50±0.02	0.67±0.00	0.60±0.05	0.50±0.01	0.56±0.05	0.51±0.06	0.52±0.07
Ellagic acid	4.63±0.04	3.18±0.04	2.87±0.04	4.22±0.35	4.31±0.30	4.79±0.38	3.56±0.23	3.26±0.12	3.41±0.06	2.94±0.24	3.38±0.09	4.08±0.04	3.02±0.23	3.84±0.34	2.74±0.07	2.16±0.06
Myricetin	0.85±0.06	0.69±0.01	0.60±0.01	0.98±0.07	0.86±0.04	0.87±0.03	0.53±0.06	0.64±0.02	0.68±0.01	0.53±0.06	0.60±0.04	0.69±0.01	0.59±0.02	0.77±0.04	0.36±0.00	0.21±0.01
Quercetin	5.50±0.31	4.68±0.06	3.50±0.03	5.34±0.24	4.68±0.15	4.28±0.11	4.02±0.48	4.73±0.14	4.37±0.29	3.50±0.17	4.76±0.11	4.48±0.22	4.09±0.20	4.43±0.16	2.67±0.11	2.17±0.14
Luteolin	0.12±0.03	0.12±0.00	0.09±0.01	0.10±0.01	0.10±0.00	0.10±0.01	0.11±0.01	0.12±0.01	0.10±0.02	0.10±0.00	0.11±0.01	0.11±0.01	0.11±0.00	0.10±0.00	0.11±0.00	0.11±0.01

Kaempferol	4.84±0.28	3.30±0.18	2.89±0.02	4.93±0.31	3.50±0.14	3.68±0.15	3.21±0.02	3.13±0.08	3.95±0.14	2.43±0.18	3.82±0.22	3.72±0.04	3.65±0.20	4.58±0.11	2.23±0.01	1.47±0.05
L-aspartic acid	2.15±0.19	2.39±0.38	2.48±1.22	3.03±0.97	3.53±1.20	4.46±1.91	3.51±1.90	2.95±0.92	3.73±1.70	3.71±2.12	4.91±2.14	3.25±1.65	3.13±2.57	2.86±0.53	2.98±0.91	2.19±0.10
L-glutamic acid	2.46±0.35	3.61±0.60	5.08±1.59	4.44±2.12	3.90±1.27	3.40±1.01	2.97±1.20	3.86±1.13	4.34±0.82	4.57±0.65	3.50±1.11	2.77 ±0.64	2.26±0.07	2.37±0.43	2.31±0.26	2.40±0.31
L-serine	0.71±0.04	0.36±0.03	0.37±0.01	0.83 ±0.22	0.51±0.35	0.68±0.03	0.99±0.12	1.07±0.25	0.85±0.12	0.79±0.10	1.04±0.06	0.84±0.18	1.15±0.16	1.00±0.09	0.75±0.07	0.44 ±0.21
glycine	0.60±0.06	0.56±0.06	0.45±0.02	0.47±0.09	0.44 ±0.02	0.79±0.01	0.57 ±0.05	0.45±0.01	0.46±0.14	0.46±0.03	0.77 ±0.09	0.45 ±0.08	0.39±0.01	0.38 ±0.01	0.42 ±0.03	0.40 ±0.02
L-histidine	1.23±0.37	0.51±0.07	0.99±0.49	0.65±0.08	1.06 ±0.48	0.95±0.11	0.74±0.01	1.19±0.85	1.01±0.42	1.00±0.68	1.19±0.24	0.74±0.31	0.45 ±0.03	0.53±0.15	0.54 ±0.04	0.57 ±0.09
L-arginine	0.95±0.31	0.60 ±0.19	1.14±0.80	1.97 ±1.47	1.65±0.33	1.06 ±0.39	1.74 ±0.37	1.19 ±0.40	0.79±0.10	1.01 ±0.49	1.36 ±0.95	0.33±0.25	1.66±0.32	1.26 ±0.71	1.21 ±0.57	1.29±1.72
L-threonine	0.26±0.08	0.51 ±0.27	0.17 ±0.06	0.16 ±0.08	0.21 ±0.16	0.96±0.24	0.54±0.15	0.43 ±0.14	0.36±0.21	0.51±0.39	0.81±0.21	0.09 ±0.08	ND	0.04±0.04	0.06±0.09	0.03±0.09
L-alanine	0.61±0.02	0.62±0.07	0.50 ±0.01	0.49±0.08	0.62±0.01	0.57±0.02	0.60±0.10	0.61±0.05	0.53±0.04	0.57 ±0.06	0.59±0.06	0.65 ±0.06	0.59 ±0.12	0.59±0.02	0.71±0.08	0.61 ±0.03
L-proline	2.61±0.25	1.70±0.05	2.22 ±0.09	2.07±0.20	2.12 ±0.02	2.23 ±0.14	2.15±0.16	3.16 ±0.03	2.49±0.12	1.95±0.22	2.46±0.30	1.81±0.13	1.76±0.15	1.54±0.17	1.50±0.07	1.52±0.14
L-Theanine	20.28±2.02	21.24±0.77	23.16±1.29	31.24 ±2.29	32.72±.079	28.29±2.51	22.76±2.40	21.26 ±0.67	19.92±1.73	18.98±0.71	18.67±0.71	17.93±0.48	17.08±1.07	12.56±2.22	12.26±2.25	10.40±0.61
L-cystine	1.28±0.11	0.62 ±0.05	0.44 ±0.13	0.25±0.01	0.28 ±0.01	0.75±0.29	0.82±0.03	0.90 ±0.03	1.00±0.61	1.35±0.08	1.25 ±0.46	1.12±0.02	0.66±0.01	1.66±0.90	0.92±0.80	0.76±0.22
L-tyrosine	1.04±.34	0.45±0.04	0.69±0.32	0.77 ±0.04	1.35 ±0.09	1.13±0.04	1.35±0.09	1.26±0.04	0.83 ±0.27	1.12 ±0.02	1.17±0.04	0.96 ±0.07	1.26±0.04	1.45±0.07	0.87±0.08	0.78±0.03
L-valine	0.41±0.02	0.35 ±0.00	0.39±0.08	0.50±0.03	0.45 ±0.02	0.49±0.11	0.43±0.01	0.44±0.00	0.47±0.12	0.50±0.01	0.55±0.03	0.34±0.03	0.32±0.03	0.35 ±0.01	0.46±0.01	0.47±0.02
L-methionine	0.57±0.04	0.66 ±0.02	0.60±0.01	0.46±0.01	0.59 ±0.04	0.49 ±0.01	0.54±0.03	0.56±0.04	0.57±0.04	0.66 ±0.02	0.60±0.01	0.46±0.01	0.59 ±0.04	0.49 ±0.01	0.54±0.03	0.56±0.04
L-isoleucine	0.85±0.06	0.58±0.03	0.87 ±0.48	0.47 ±0.02	0.82±0.05	0.49±0.15	0.81±0.04	0.77±0.03	0.85±0.06	0.58±0.03	0.87 ±0.48	0.47 ±0.02	0.82±0.05	0.49±0.15	0.81±0.04	0.77±0.03
L-leucine	0.64 ±0.21	0.98±0.03	0.99±0.04	0.51±0.01	0.80 ±0.04	0.65 ±0.06	0.79±0.03	0.77±0.03	0.64 ±0.21	0.98±0.03	0.99±0.04	0.51±0.01	0.80 ±0.04	0.65 ±0.06	0.79±0.03	0.77±0.03
L-phenylalanine	0.85 ±0.01	0.68 ±0.02	1.35 ±0.42	0.90±0.03	1.54±0.07	1.28±0.42	1.75 ±0.10	1.31 ±0.05	0.85 ±0.01	0.68 ±0.02	1.35 ±0.42	0.90±0.03	1.54±0.07	1.28±0.42	1.75 ±0.10	1.31 ±0.05
L-lysine	0.67±0.01	0.61±0.01	0.83 ±0.07	0.57±0.01	0.75 ±0.02	0.68 ±0.05	0.77±0.02	0.71 ±0.02	0.67±0.01	0.61±0.01	0.83 ±0.07	0.57±0.01	0.75 ±0.02	0.68 ±0.05	0.77±0.02	0.71 ±0.02
TFs	0.08±0.01	0.10±0.01	0.10±0.00	0.11±0.01	0.09±0.01	0.07±0.01	0.09±0.00	0.08±0.01	0.09±0.00	0.11±0.01	0.11±0.01	0.08±0.01	0.08±0.00	0.08±0.01	0.07±0.01	0.08±0.00
TBs	4.68±0.02	5.90±0.02	5.31±0.03	5.25±0.06	5.14±0.11	3.64±0.02	3.07±0.09	4.50±0.10	4.78±0.06	4.85±0.04	4.54±0.04	4.93±0.09	5.69±0.10	4.78±0.09	3.61±0.10	3.58±0.12
TRs	1.81±0.2	2.19±0.05	2.28±0.02	2.31±0.02	2.70±0.01	2.73±0.09	2.78±0.04	2.89±0.02	2.920±0.04	2.97±0.06	2.96±0.05	3.01±0.02	3.00±0.02	3.08±0.07	3.45±0.05	3.48±0.04
Chlorophyll-a	2.36±0.01	3.21±0.03	2.35±0.02	2.47±0.02	2.37±0.01	1.83±0.01	2.07±0.01	2.85±0.00	2.56±0.00	2.00±0.00	1.88±0.13	2.31±0.01	3.08±0.01	2.13±0.01	1.50±0.05	1.56±0.00
Chlorophyll-b	0.94±0.04	0.56±0.01	0.54±0.05	0.37±0.02	0.23±0.03	0.26±0.04	0.23±0.03	0.37±0.02	0.35±0.01	0.35±0.03	0.20±0.02	0.24±0.04	0.35±0.03	0.20±0.03	0.22±0.14	0.25±0.02
Carotenoids	0.68±0.01	0.79±0.01	0.71±0.02	0.56±0.01	0.44±0.01	0.34±0.01	0.37±0.00	0.54±0.01	0.56±0.00	0.27±0.01	0.28±0.02	0.37±0.01	0.43±0.01	0.33±0.01	0.17±0.03	0.23±0.00

Anthocyanin	3.73±0.00	3.90±0.29	3.90±0.29	4.07±0.29	3.73±0.29	3.56±0.29	3.39±0.29	3.29±0.29	3.05±0.29	3.39±0.29	3.22±0.00	2.89±0.29	3.22±0.29	2.21±0.29	2.38±0.29	2.55±0.29

Table S3. The correlation coefficient between 20 functional compounds in organic green tea and storage years.

Pearson'r	Storage years
Storage years	1
Tea polyphenols	-.0945**
Soluble sugars	-.0588*
Total amino acids	-.0648**
Ellagic acid	-.0510*
Gallic acid	0.779**
EGC	-0.863**
GCG	-0.500*
CG	0.596*
Luteoloside	-0.854**
Myricetin	-0.639**
Quercetin	-0.618*
L-glutamic acid	-0.527*
L-proline	-0.505*
Theanine	0-.748**
L-methionine	-0.613*
L-leucine	-0.567*
TBs	0.949**
Chlorophyll-b	-0.685**
Carotenoids	-0.815**
Anthocyanin	-0.904**

**. $p<0.01$, *. $p<0.05$, Significant correlation.

Table S4. The correlation coefficient between 18 functional compounds in organic green tea and electronic tongue signals.

Pearson'r	SRS	GPS	STS	UMS	SPS	SWS	BRS
Tea polyphenols	0.868**	0.845**	0.866**	0.916**	0.949**	0.709**	0.896**
Soluble sugars	0.391	0.310	0.275	0.650**	0.635**	0.056	0.481
Total amino acids	0.458	0.424	0.403	0.661**	0.696**	0.144	0.577*
Gallic acid	-.608*	-.581*	-.632**	-.883**	-.820**	-0.457	-0.647**
EGC	0.787**	0.757**	0.763**	0.744**	0.848**	0.614*	0.766**
CG	-0.425	-0.374	-0.368	-0.518*	-0.617*	-0.127	-0.497
Luteoloside	0.953**	0.968**	0.943**	0.691**	0.813**	0.934**	0.875**
Myricetin	0.529*	0.541*	0.560*	0.725**	0.666**	0.386	0.566*
Quercetin	0.501*	0.506*	0.556*	0.739**	0.657**	0.474	0.497
L-glutamic acid	0.355	0.279	0.359	0.643**	0.542*	-0.005	0.477
L-proline	0.324	0.243	0.293	0.632**	0.561*	0.099	0.276
Theanine	00.588*	00.552*	0.537*	0.709**	0.775**	0.248	0.704**
L-methionine	0.640**	0.598*	0.585*	0.596*	0.621*	0.578*	0.619*
L-leucine	00.517*	0.468	0.477	0.596*	0.605*	0.354	0.530*
TBs	-0.937**	-0.930**	-0.975**	-0.883**	-0.929**	-0.790**	-0.905**
Chlorophyll-b	0.771**	0.767**	0.831**	0.616*	0.643**	0.772**	0.641**
Carotenoids	0.810**	0.800**	0.855**	0.837**	0.818**	0.728**	0.774**
Anthocyanin	0.807**	0.772**	0.807**	0.830**	0.911**	0.550*	0.856**

**. p<0.01, *. p<0.05, Significant correlation.

Table S5. The correlation coefficient between the color difference values of dry tea and tea liquor and tea pigments.

Pearson'r	L*(Dry tea)	a*(Dry tea)	b*(Dry tea)	LL*(Tea liquor)	La*(Tea liquor)	Lb*(Tea liquor)
TFs	-0.221	-0.289	0.506*	0.500*	-0.559*	0.394
TRs	0.047	-0.165	0.164	0.208	-0.453	0.300
TBs	-0.536*	0.677**	-0.623**	-0.407	0.645**	-0.084
Chlorophyll-a	0.242	-0.251	0.222	0.256	-0.414	0.235
Chlorophyll-b	0.340	-0.549*	0.534*	0.488	-0.537*	0.360
Carotenoids	0.417	-0.535*	0.439	0.410	-0.598*	0.249
Anthocyanin	0.558*	-0.741**	0.603*	0.449	-0.812**	0.075

**. p<0.01, *. p<0.05, Significant correlation.

Table S6. BPNN classification results and model performance metrics based on E-tongue, chromatism, and 10 compounds (VIP≥1).

Date sources	Neural model	Training set accuracy	Test set accuracy	Recall	Precision	F1-score
E-tongue	7-8-3	100%	97.92%	0.9793	0.9723	0.9758
10 compounds (VIP≥1)	10-9-3	100%	91.67%	0.9443	0.9167	0.9303
Chromatism	6-5-3	99.07%	94.44%	0.9583	0.9523	0.9553

Table S7. The correlation coefficient between the antioxidative activity and functional components of organic green tea.

Pearson'r	DPPH	SSA	HSA	ABTS	FRAP
Caffeine	0.398	-0.232	-0.262	0.238	-0.057
Gallic acid	-0.170	-0.359	-0.676**	0.497	-0.310
GC	-0.175	0.444	.568*	-0.060	0.094
EGC	-0.244	0.491	0.869**	-0.562*	0.429
C	0.017	0.535*	0.344	0.089	0.043
EGCG	0.097	0.051	0.148	0.028	-0.097
EC	0.109	0.522*	0.498*	-0.068	0.154
GCG	-0.055	0.324	0.590*	-0.290	0.139
ECG	0.097	0.221	0.125	0.256	-0.172
CG	-0.011	-0.428	-0.448	0.215	-0.245
Taxifolin	-0.250	0.435	0.216	-0.474	0.350
Luteoloside	-0.574*	0.512*	0.795**	-0.717**	0.574*
Rutin	0.266	-0.011	0.190	0.331	-0.254
Ellagic acid	-0.186	0.329	0.464	-0.136	0.054
Myricetin	-0.084	0.246	0.463	-0.379	0.207
Quercetin	0.100	0.328	0.465	-0.255	0.026
Luteolin	-0.043	0.508*	0.101	0.116	-0.203
Kaempferol	0.169	0.354	0.318	-0.176	-0.046
L-aspartic acid	0.351	-0.155	-0.086	0.378	-0.293
L-glutamic acid	0.095	-0.179	0.406	-0.643**	0.588*
L-serine	0.795**	0.105	-0.390	0.533*	-0.585*
glycine	-0.206	0.429	0.424	0.042	0.018
L-histidine	0.063	0.113	0.489	-0.312	0.333
L-arginine	0.071	0.325	-0.277	0.120	0.044
L-threonine	-0.040	0.220	0.349	-0.005	0.126
L-alanine	-0.223	-0.058	-0.233	0.549*	-0.419
L-proline	0.222	0.257	0.545*	-0.296	0.268
Theanine	-0.237	0.186	0.567*	-0.462	0.425
L-cystine	0.395	-0.017	-0.305	0.339	-0.334
L-tyrosine	0.412	0.039	-0.354	0.450	-0.331
L-valine	-0.096	0.102	0.001	0.061	0.208
L-methionine	-0.260	0.080	0.580*	-0.546*	0.524*
L-isoleucine	0.153	0.237	0.400	-0.346	0.346
L-leucine	0.006	-0.050	0.484	-0.452	0.373
L-phenylalanine	0.310	0.044	0.139	0.077	0.074
L-lysine	0.202	0.057	0.409	-0.284	0.250
Tea polyphenols	-0.241	0.447	0.850**	-0.713**	0.577*
Total flavonoids	-0.188	-0.046	0.267	-0.122	-0.007
Soluble sugars	-0.002	0.105	0.515*	-0.360	0.443

Total amino acids	-0.077	0.143	0.481	-0.324	0.280
TFs	0.124	0.021	0.202	-0.465	0.305
TRs	0.181	-0.307	0.115	-0.423	0.086
TBs	0.299	-.541*	-0.792**	0.767**	-.558*
Chlorophyll-a	0.199	-0.036	0.297	-0.398	0.089
Chlorophyll-b	-0.306	0.528*	0.660**	-0.749**	0.554*
Carotenoids	-0.169	0.252	0.730**	-0.770**	0.525*
Anthocyanin	-0.244	0.350	0.692**	-0.708**	0.550*

**. $p<0.01$, *. $p<0.05$, Significant correlation.
